# Dietary Fatty Acids Alter Lipid Profiles and Induce Myocardial Dysfunction without Causing Metabolic Disorders in Mice

**DOI:** 10.3390/nu10010106

**Published:** 2018-01-19

**Authors:** Bainian Chen, Yifan Huang, Dong Zheng, Rui Ni, Mark A. Bernards

**Affiliations:** 1 Institutes of Biology and Medical Sciences, Soochow University, Suzhou 215000, China; bainianchen@yahoo.com (B.C.); zhengdong1225@163.com or dzheng25@uwo.ca (D.Z.); 2 Lawson Health Research Institute, London Health Sciences Centre, London, ON N6A 4G5, Canada; rni2@uwo.ca; 3 Department of Medicine, University of Western Ontario, London, ON N6A 5A5, Canada; 4 Department of Pathology, University of Western Ontario, London, ON N6A 5C1, Canada; 5 Department of Biology, University of Western Ontario, London, ON N6A 2B7, Canada; bernards@uwo.ca

**Keywords:** diet, lipid profiles, myocardial dysfunction, saturated fatty acids

## Abstract

Oversupply of bulk saturated fatty acids (SFA) induces metabolic disorders and myocardial dysfunction. We investigated whether, without causing metabolic disorders, the uptake of individual dietary SFA species alters lipid profiles and induces myocardial dysfunction. C57BL/6 mice were fed various customized long-chain SFA diets (40% caloric intake from SFA), including a beef tallow (HBD), cocoa butter (HCD), milk fat (HMD) and palm oil diet (HPD), for 6 months. An isocaloric fat diet, containing medium-chain triglycerides, served as a control (CHD). Long-term intake of dietary long-chain SFA differentially affected the fatty acid composition in cardiac phospholipids. All long-chain SFA diets increased the levels of arachidonic acid and total SFA in cardiac phospholipids. The preferential incorporation of individual SFA into the cardiac phospholipid fraction was dependent on the dietary SFA species. Cardiac ceramide content was elevated in all mice fed long-chain SFA diets, while cardiac hypertrophy was only presented in mice fed HMD or HPD. We have demonstrated that the intake of long-chain SFA species differentially alters cardiac lipid profiles and induces cardiac dysfunction, without causing remarkable metabolic disorders.

## 1. Introduction

Obesity and type-2 diabetes are prevalent in Western countries and countries elsewhere that have adopted a Western lifestyle. In both conditions, lipotoxic cardiomyopathy commonly ensues, due to excessive fatty acid uptake by cardiomyocytes. The outcomes of patients with lipotoxic cardiomyopathy include severe heart failure, leading to death or heart transplantation, representing a major health problem. Cumulative evidence indicates that high-fat diets induce an elevation of blood triglycerides and free fatty acids, and persistent accumulation of lipids in the heart, leading to myocardial dysfunction, also described as lipotoxic cardiomyopathy [1,2]. Cardiac dysfunction, associated with abnormal fat storage in cardiomyocytes is observed in animal models of obesity [3,4,5]. In addition, cardiomyocytes, isolated from high-fat fed mice, displayed contractile defects, compared with those from control animals [6]. In these animal models of obesity, myocardial dysfunction may be directly associated with saturated fatty acid (SFA) oversupply. Indeed, previous investigations have shown that oversupply of SFA directly impairs contractile performance and induces apoptosis of cardiac myocytes, in vitro [7,8,9]. However, whether these effects are mediated by bulk SFA or by individual lipid species in vivo remains largely elusive.

The predominant SFA in Western diets are myristic acid (14:0), palmitic acid (16:0) and stearic acid (18:0), which display different metabolic properties [10]. It has been recently reported that mice fed a high palmitate diet develop cardiac contractile dysfunction [11,12], and a high milk fat-based diet induces obesity, myocardial hypertrophy and dysfunction in mice, which may be due to its constituent SFA, myristate [13]. These studies suggest a potential effect of individual dietary lipid species on the compromising of myocardial function. However, cardiac changes resulting from SFA oversupply may be secondary to the complications associated with obesity and metabolic disorders. Thus, the impact of individual dietary lipid species on the heart remains incompletely understood.

In this study, we hypothesized that individual dietary SFA species may directly induce differential changes in lipid profiles and myocardial dysfunction. Accordingly, we investigated whether the oversupply of individual SFA species, originating from commonly-used Western diet fats, including beef tallow, cocoa butter, milk fat and palm oil, could induce cardiac dysfunction and alter lipid profiles in mice, without causing metabolic disorders.

## 2. Materials and Methods

### 2.1. Animals and Diets

This study conforms to the Guide for the Care and Use of Laboratory Animals, published by the US National Institutes of Health (NIH Publication No. 85-23). All experimental procedures were approved by the Animal Use Subcommittee at the University of Western Ontario, Canada (Ethical Approval Code: 2008-079). Breeding pairs of C57BL/6 mice were purchased from The Jackson Laboratory (Bar Harbor, ME, USA). All animals were kept under controlled conditions at room temperature and constant relative humidity, with free access to food and water. The customized high-fat diets with major forms of long-chain SFA, including a high beef tallow diet (HBD), high cocoa butter diet (HCD), high milk fat diet (HMD) and high palm oil diet (HPD), representing diets rich in stearic acid (HBD and HCD), myristic acid (HMD) and palmitic acid (HPD), respectively, were all purchased from Harlan Laboratories. An isocaloric high-fat diet (CHD) was designed to contain medium-chain triglycerides as a control. The contents (% by weight) of protein, carbohydrate and fat in these high-fat diets were 20.9%, 44.8% and 20.2%, respectively. The calculated caloric intakes from these nutrients (% kcal) were 18.8%, 40.3% and 40.9%, respectively. The detailed fatty acid compositions of the high-fat diets are summarized in Table S1 of Supplementary File 2. All the high SFA diets were stored at −20 °C and supplied fresh to animals every 2–3 days.

In our preliminary experiment, of 6-month duration, there was no difference in cardiac function indices and metabolic parameters (including body weight, heart weight, blood pressure and blood glucose level) between mice fed a normal rodent diet (9% fat, *w*/*w*) and mice fed the control high-fat diet (CHD, 20% fat, *w*/*w*). Thus, we did not include a normal diet group in the current study, and all the results from dietary long-chain SFA groups were compared with those from the isocaloric control CHD group.

Male C57BL/6 mice (aged 4–6 weeks, *n* = 4–5/group) were fed experimental diets for 3 or 6 months. Animal body weight was measured weekly among the 6-month diet groups. Mice were fasted overnight before terminal sample collection. Blood and heart tissues were collected immediately after animals were euthanized by cervical dislocation. Heart weight was recorded prior to the tissue being frozen at −80 °C. The serum was isolated and kept at −80 °C.

### 2.2. Statistical Analysis

A quantitative analysis for fatty acids and ceramides was based on the calibration curves of internal standards from a linear regression model. The peak area of each sample was normalized to the area of its respective internal standard, and either volume of serum samples or mass of heart samples extracted. All data were given as mean ± standard deviation, and analyzed with a one-way ANOVA followed by a Newman–Keuls test. A two-tailed value of *p* < 0.05 was considered statistically significant.

Other detailed methods are available in online Supplementary File 1.

## 3. Results

### 3.1. Metabolic Characteristicsin Mice Fed High SFA Diets

**Figure 1:**
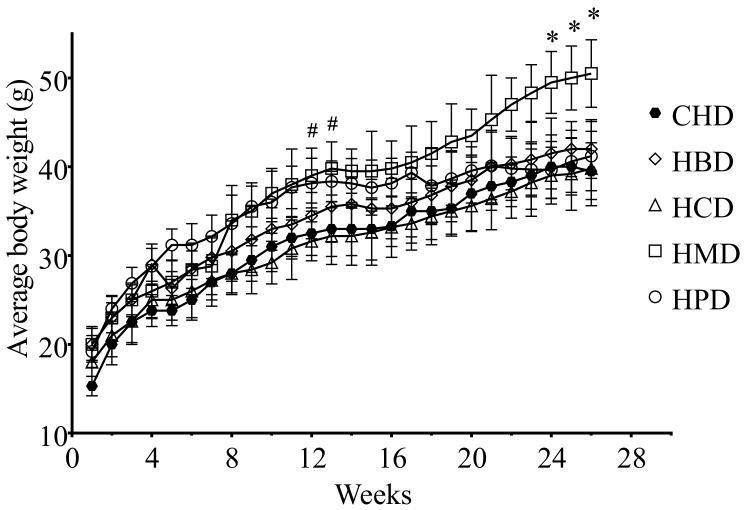
Body weight gains of 6-month diet mice. Each dot and error bar represent the mean value and standard deviation from four to five animals. * *p* < 0.05, HMD versus CHD; # *p* < 0.05, HPD versus CHD. CHD, control high fat diet; HBD, high beef tallow diet; HCD, high cocoa butter diet; HMD, high milk fat diet; HPD, high palm oil diet.

There were no significant differences in heart weight and heart/body weight ratio among the different high SFA diets groups, including CHD (Table S2 of Supplementary File 2). However, the body weight of the HPD group was heavier than that of the CHD group after 3 months, whereas the HMD resulted in an increased body weight, compared with CHD, after a longer feeding period (6 months) (Figure 1).

**Figure 2:**
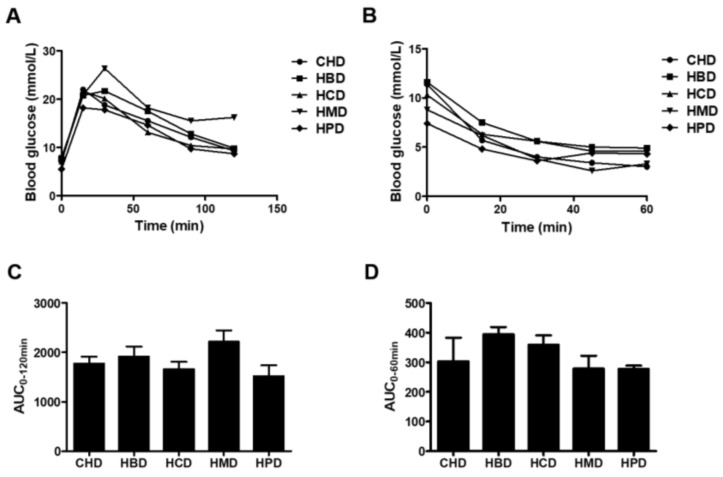
Blood glucose responses to intraperitoneal glucose tolerance (IPGTT) and insulin tolerance (IPITT) tests. (**A**) Blood glucose concentrations for IPGTT and (**B**) blood glucose concentrations for IPITT. Each dot represents the mean value from four to five animals; (**C**,**D**) Area under the curves (AUC) for blood glucose during IPGTT and IPITT, respectively. Data are presented as mean ± standard deviation, *n* = 4–5.

No significant changes in either glucose tolerance or insulin tolerance were observed among any of the high SFA diets groups (Figure 2). However, HMD induced a slight increase in the area under curve (AUC) for intraperitoneal glucose tolerance (IPGTT), compared with that of CHD (2217.4 ± 460.0 versus 1770.2 ± 288.1), indicating HMD may contribute to impaired glucose tolerance in mice. There were no differences in systolic/diastolic blood pressure, heart rate and fasting blood glucose concentration in any of the studied groups (Table S3 of Supplementary File 2). In short, except for body weight between some groups, at some stages, there were no changes in almost all main indicators of metabolic disorder among groups.

### 3.2. Cardiomyocyte Cross-Sectional Area in Mice Fed High SFA Diets

**Figure 3:**
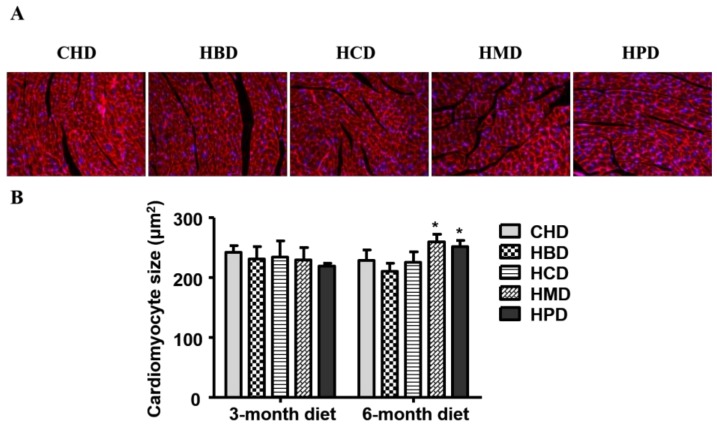
Histological analysis of cardiac tissue from mice fed high-fat diets. (**A**) Representative fluorescent images for cardiac tissue section from mice fed high-fat diets for six months (magnification at 200×); (**B**) Quantitative analysis of cardiomyocyte cross-sectional area. Data are presented as mean ± standard deviation, *n* = 3–5 mouse hearts in each group. * *p* < 0.05, compared with CHD group of 6-month diet).

In this study, three months of high-fat diet feeding did not result in cardiac hypertrophy, as the cardiomyocyte size was comparable among all the experimental groups (Figure 3) at this time point. While six months of high fat diet feeding using HBD or HCD did not result in an enlarged cardiomyocyte size (210.24 ± 13.37 and 225.74 ± 17.07, respectively), the cross-sectional area of cardiomyocytes increased in the HMD and HPD groups (259.18 ± 12.53 and 251.58 ± 9.73, respectively), compared with the CHD group (228.56 ± 17.07).

### 3.3. Cardiac Function in Mice Fed High SFA Diets

**Figure 4:**
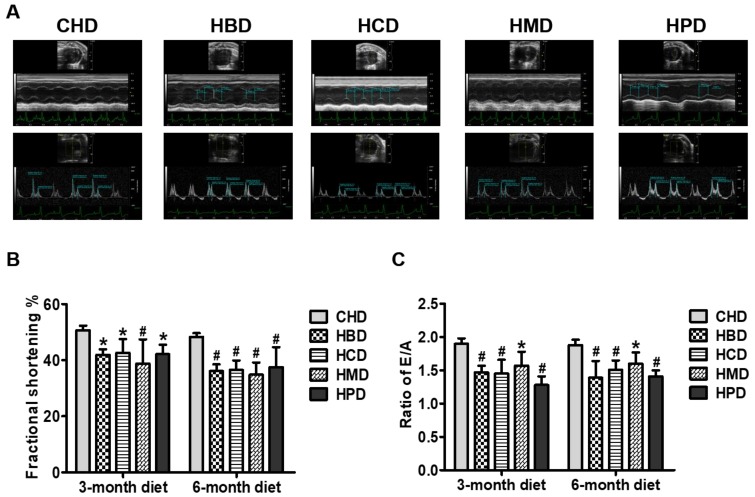
Cardiac function in mice fed high-fat diets. (**A**) Representative echocardiographic images obtained from mice fed 6-month high-fat diets; (**B**) Quantification results of fractional shortening, based on echocardiograms performed at three and six months into the feeding period; (**C**) Quantification results of the ratio of the peak velocity of early to late filling of mitral inflow (E/A), at the indicated time points. Data are presented as mean ± standard deviation, *n* = 4–7. * *p* < 0.05 and # *p* < 0.01 versus CHD.

As shown in Figure 4A, fractional shortening was significantly decreased in all the long-chain SFA diet groups, compared with the CHD group, at both 3 and 6 months into the feeding regimen, suggesting that high intake of these dietary long-chain SFA (beef, milk, cocoa and palm oil) induces cardiac systolic dysfunction in mice. Determination of the ratio of mitral inflow (E/A) revealed marked deterioration of cardiac diastolic performance in mice fed high long-chain SFA diets for 3 months and onward (Figure 4B). The other relevant parameters for cardiac function are shown in Table S4 of Supplementary File 2.

### 3.4. Fatty Acid Profile of Serum from Mice Fed High-Fat Diets

In the present study, fatty acid profiles of total lipids in serum from mice fed different dietary SFA were analyzed by gas chromatography (Table 1). An increased level of 14:0 was observed in 3-month HMD-fed mice, compared with the CHD mice (0.15 ± 0.01 versus 0.09 ± 0.02, *p* < 0.01), while a significant decrease of 14:0 content was observed in HCD and HPD groups, compared with the CHD group. HPD induced a marked increase of 16:0 in serum lipids, whereas HBD-, HCD- and HPD-fed mice showed elevated 18:0 levels compared with CHD-fed mice. These alterations of individual lipid species in serum reflect the intake of constituent SFA in diets. However, the total SFA content was much higher, only in the HPD group, in comparison to the CHD group (at both three months and six months).

As for monounsaturated fatty acids (MUFA), the level of palmitoleic acid (16:1) was 20–50% lower in both HCD and HPD groups, while the levels of oleic acid (18:1) were about 30% higher in both HBD and HMD groups, compared with the CHD group (Table 1). The most abundant polyunsaturated fatty acids (PUFA) detected in the serum, arachidonic acid (20:4, AA), accumulated much more in serum lipids from mice fed HBD, HCD and HPD for 6 months, than those from mice fed CHD (*p* < 0.05 or *p* < 0.01). Interestingly, the levels of docosahexaenoic acid (22:6, DHA) were greatly lower in mice fed CHD for six months, compared with those fed CHD for three months (0.25 ± 0.06 versus 0.44 ± 0.06), while slight changes in DHA level in serum lipids were documented in other high dietary fat diets, when a comparison was made between medium (3 months) and long (6 months) feeding periods. This might suggest that long-term intake of medium-chain SFA led to elevated transformation of DHA to other metabolites.

### 3.5. Fatty Acid Profile of Neutral Lipids and Phospholipids in Heart Tissues from Mice Fed High SFA Diets

To examine whether dietary SFA induced alterations in fatty acid composition in cardiac tissues, we extracted total fats from hearts of mice fed different high-fat diets and fractionated them into neutral lipids and phospholipids. The quantitative results for fatty acid profiles from neutral lipids and phospholipids are summarized in Table 2 and Table 3, respectively. The quantification of fatty acids originating from neutral lipids revealed that feeding HMD for three months significantly increased the quantity of SFA species, including 14:0, arachidic acid (20:0) and docosanoic acid (22:0), and that feeding HMD for six months led to a significant increase in 18:0 (Table 2). Furthermore, feeding with HBD, HCD and HMD for six months caused a notable decrease in DHA in mice, compared with CHD. Intriguingly, most of the other components in the fatty acid profile of neutral lipids showed no significant differences among all the high SFA diet groups. As triglycerides are one of the major forms of neutral lipids in mammalian cells [14], our results suggest that the dietary fats included in the present study had little effect on the fatty acid composition of triglycerides.

Phospholipids are one of the most abundant membrane lipid classes and play multiple pivotal roles in cell membrane function under physiological and pathological conditions. Early reports have illustrated that the fatty acid composition of phospholipids changes in response to dietary fat intake [15,16,17]. Our results for the quantitative analysis of fatty acids from cardiac phospholipids showed that feeding on both HBD and HMD significantly increased the level of 14:0 in phospholipids, while feeding on both HMD and HPD increased the level of 16:0 in phospholipids in mice fed high-fat diets for three months, compared with CHD (Table 3). In addition, the concentration of 16:0 in phospholipids from animals fed a 6-month diet was significantly elevated in the HPD group, compared with the CHD group. All dietary high SFA diets tested led to markedly increased 18:0 levels, in comparison to CHD, especially after six months of feeding. Furthermore, the concentration of 20:0 was higher in cardiac phospholipids from mice fed HCD for both three and six months, and HMD for three months, than those from the CHD group. The total SFA contents in cardiac phospholipids from mice fed HBD, HCD, HMD and HPD for 3 months were 14.69 ± 0.89, 15.49 ± 0.74, 16.22 ± 0.59 and 12.75 ± 0.73, respectively, all of which were significantly higher than that of CHD-fed mice (11.32 ± 0.28). The same pattern of total SFA changes was also observed in 6-month feeding animals (Table 3), which indicated that more SFA were incorporated into the phospholipid fraction of cardiac tissues, due to long-term high intake of dietary SFA.

For MUFA, all the dietary fat diets resulted in a significant reduction in the level of eicosenoic acid (20:1) in cardiac phospholipid fractions from mice fed high SFA diets for three and six months, compared with those of CHD. Moreover, the level of 16:1 was also decreased in mice fed HPD for three and six months. As for PUFA, consumption of HMD for three and six months resulted in a significant increase in docosapentaenoic acid (22:5), whereas consumption of HPD for three months enhanced the level of 22:5 compared with CHD (Table 3). There was no change in DHA content in the phospholipid fraction of hearts among all the diet groups. However, a marked increase in the level of AA from cardiac phospholipids was observed in all high SFA diets groups, compared with the CHD group, especially when animals were fed with these diets for six months.

### 3.6. Ceramide Content in Heart Tissues from Mice Fed High SFA Diets

**Figure 5:**
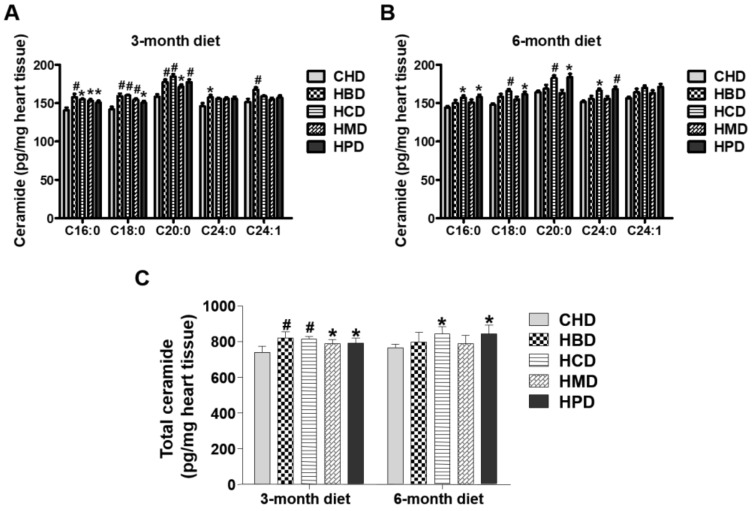
Ceramide levels in cardiac tissue of mice fed high-fat diets. (**A**,**B**) Concentrations of individual ceramide species in the hearts of mice fed high-fat diets for three and six months, respectively; (**C**) Quantitative results of total ceramide in cardiac tissue of mice fed high-fat diets. Data are presented as mean ± standard deviation, *n* = 4–8. * *p* < 0.05 and # *p* < 0.01 versus CHD.

In this study, levels of several individual ceramide species (C16:0, C18:0 and C20:0) in heart tissues were slightly increased in response to feeding HBD, HCD, HMD and HPD for three months, compared with those in the CHD group (Figure 5). The total ceramide content (calculated as the sum of ceramide species) was also higher in all the high-fat diet groups, compared with that of CHD group. For longer chain ceramide species (C24:0 and C24:1), the increased level was only observed in the HBD group (Figure 5). However, when mice were fed high-fat diets for six months, the high-fat induced accumulation of ceramides (both total and individual ceramide) was not significant in the HBD and HMD groups, in comparison to the CHD group. Only HCD and HPD caused an increase in cardiac ceramide content, compared with CHD, during the 6-month feeding regimen.

### 3.7. Malondialdehyde Level in Heart Tissues from Mice Fed High SFA Diets

**Figure 6:**
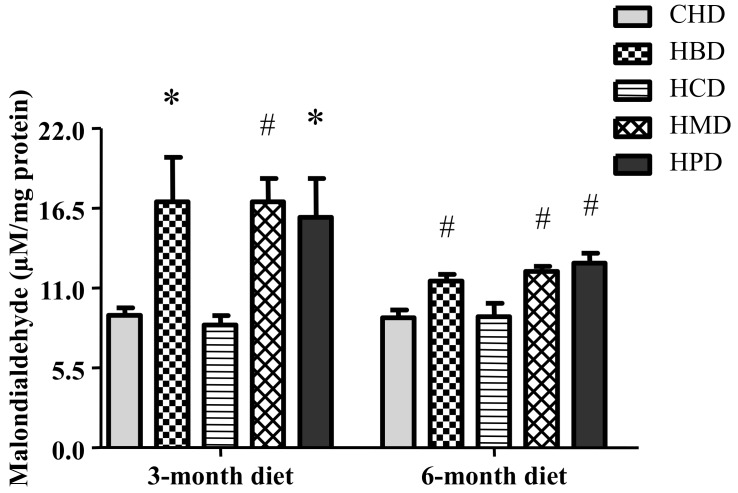
Malondialdehyde level in cardiac tissue of mice fed high-fat diets. Data are presented as mean ± standard deviation, *n* = 4–8. * *p* < 0.05 and # *p* < 0.01 versus CHD.

Levels of malondialdehyde in heart tissues were significantly increased in response to feeding HBD, HMD and HPD for three months and six months (Figure 6). However, HCD did not increase the levels of malondialdehyde in heart tissues.

## 4. Discussion

The major findings of this study are that uptakes of all dietary long-chain SFA induced cardiac dysfunction, without causing significant changes in blood pressure, fasting blood glucose, glucose tolerance and insulin resistance in mice, suggesting that over-supply of individual SFA may compromise myocardial function by mechanisms independent of the majority of metabolic disorders. The changes in blood fatty acid profile, altered fatty acid composition in cardiac phospholipids and increased cardiac ceramide and malondialdehyde contents, induced by varied SFA consumption, highlight the potential mechanisms by which dietary SFA intake compromised cardiac function. Thus, this study lays the foundation for future studies about the influence of dietary SFA intake on the heart. To the best of our knowledge, this is the first study that has comprehensively investigated the effects of various SFA diets intake on the heart in vivo.

### 4.1. Design of Dietary Groups and Metabolic Profiles

It has been recognized that the type of dietary fats consumed in a diet may be a key determinant for heart health [18,19]. SFA, the most dominant fats in Western food, are thought to be harmful to the cardiovascular system when over-consumed [20]. An over-supply of SFA may cause metabolic disorders, which contribute to cardiovascular diseases. Intakes of SFA may also induce direct damage to the heart.

In this study, we examined several SFAs commonly found in the diets of our population, including palm oil (HPD), milk fat (HMD), beef tallow (HBD) and cocoa butter (HCD), which represent diets rich in myristic acid (HMD), palmitic acid (HPD), and stearic acid (HBD and HCD), respectively. Because we did not see any differences between CHD and normal diet, in terms of body weight, blood glucose, blood pressure, cardiomyocyte size and myocardial function, in our preliminary experiments, we used CHD as a control diet for this study. As a control diet, CHD only contained short- to medium-chain SFA (6:0 to 10:0), whereas the major forms of SFA in HBD, HCD, HMD and HPD were long-chain SFA (14:0 to 18:0). Notably, we chose the total fat content of high SFA diets with 20% (*w*/*w*), or calculated as about 40% energy from fat, because they altered blood lipid profiles but did not change most vital metabolic profiles, including body weight, blood pressure level, fasting blood glucose level, insulin and glucose tolerance. Thus, these SFA diets allow us to investigate the link between individual SFA oversupply and cardiac changes, without the complications associated with metabolic disorders. It is should be noted that there were two exceptions to the lack of changes in body weight noted above. Specifically, after three months of HPD feeding as well as six months HMD feeding, significant increases in the body weight of mice were noted. The difference in body weight between HPD and CHD groups diminished after six months feeding, hence, so far, it is still unclear whether HPD affected the body weight of mice. Nevertheless, the increased body weight induced by HMD feeding was consistent with a previous study in which mice fed a Western diet, containing 21% milk fat, gained 25–35% more body weight than control mice [21].

### 4.2. SFA Intakesand Cardiac Abnormalities

Compared with the CHD group, the four high dietary long-chain SFA groups had obvious cardiac dysfunction. Since CHD only contained short- to medium-chain SFA (6:0 to 10:0), whereas the major forms of SFA in other high dietary SFA groups were long-chain SFA (14:0 to 18:0), the intake of long-chain SFA induced cardiac dysfunction independent of the SFA species.

High-fat diets have been implicated in cardiac hypertrophy in several animal-based studies [13,22,23]. It is currently unknown whether the varied composition of fatty acids leads to distinct hypertrophy in the heart. A recent study has revealed that a milk fat-based diet led to significant left ventricular hypertrophy in mice [13], which was due to the uptake of myristic acid but not palmitic acid. Our data demonstrated that only long-term feeding of HMD and HPD diets induced cardiac hypertrophy, suggesting that long-term intake of both high myristic acid and palmitic acid may promote the development of cardiac hypertrophy. Thus, the incidence and development of cardiac hypertrophy may be dependent of the type and composition of fatty acids in diets.

### 4.3. SFA Intakes and Myocardialphospholipids Alteration

Considering that the type and level of blood fatty acids are closely associated with the cardiac fatty acid composition, the investigation of fatty acid profile changes in heart tissues provided useful data for further analyzing the effect of the SFA diet on cardiac function. Previous studies have demonstrated that modulation of membrane fatty acid composition in the heart by dietary fats may affect membrane fluidity or permeability, phase behavior and membrane fusion, leading to the perturbations in membrane-bound enzymes and receptors, which ultimately impacts cardiac function [4,24,25]. In addition, dietary fat may also change cardiac mitochondrial phospholipid fatty acid side chain composition [26]. Neutral lipids, which mostly consist of triglycerides in mammalian cells, represent tissue lipid storage; while phospholipids, the most abundant lipid class in biomembranes, influence membrane properties and function [14,25]. Therefore, we measured the fatty acid profiles of neutral lipids and phospholipids in heart tissues. We observed that total cardiac SFA content and cardiac phospholipid composition, rather than neutral lipids, were more prone to be influenced by dietary SFA diet consumption. Our results also indicated that the increased amounts of SFA in cardiac phospholipids were mainly dependent on dietary fatty acid types. Previous studies have shown that an increase in the saturation of membrane phospholipids can impair heart function by reducing cardiac membrane lipid fluidity [25]. In addition, the elevated levels of SFA in cardiac membrane lipids enhanced myocardial oxygen consumption and reduced contractile function during post-ischemic recovery in mice [4]. Taken all together, we conclude that the accumulation of SFA in cardiac membrane phospholipids may be important in dietary SFA intake-induced cardiac dysfunction.

Another interesting finding is that after a 6-month feeding, all SFA diets resulted in a significantly increased content of AA in the cardiac phospholipid fraction. AA belongs to the n-6 PUFA family and is a key substrate for the synthesis of eicosanoids, including prostaglandins, thromboxanes and leukotrienes, which exhibit pro-inflammatory activity in various types of cells [27]. Numerous data suggest that myocardial inflammation, activated by unhealthy diets, is closely associated with the incidence and development of cardiovascular diseases [28]. It is possible that increased AA may compromise cardiac function. However, we noticed that cardiac linoleic acid (18:2), a primary precursor of AA, was lower after 6-month feeding among all SFA groups, suggesting a possible unconfirmed metabolic pathway responsible for the increased generation of AA. To a certain degree, changes in AA and linoleic acid in our study are inconsistent with the study performed by O’Connell et al. [26], which suggested that, compared with low SFA diets, high SFA diets decrease cardiac subsarcolemmal mitochondrial linoleic acid, without causing significant alterations in AA. Differences in the fatty acid composition of diets, feeding duration and control group probably contribute to the inconsistencies between studies. So far, we cannot exclude that other potential mechanisms are at play; therefore, more evidence would be helpful to clarify them in future.

### 4.4. SFA Intakes, Myocardial Ceramides and Malondialdehyde

Ceramides, one of the component lipids of cell membranes, are thought to be cardiac lipotoxic, and accumulate in the hearts of animals fed high-fat diets. Accumulation of ceramides has been implicated in apoptotic cell death in cardiomyocytes [5,12] and thus may contribute to cardiac dysfunction. Although cardiac inflammation and apoptosis, induced by elevated ceramide levels, may promote the development of myocyte hypertrophy [23,29,30], we did not observe the corresponding enlarged cardiomyocyte size in any diet group, except for HMD and HPD, at the end of 6-month diet feeding. This suggests that increased ceramide levels are either not associated with, or not sufficient to induce, cardiac hypertrophy, in mice fed HMD and HPD diets.

As a product of the degradation of polyunsaturated lipids, malondialdehyde is a typical indicator of oxidative stress. Significant increases in malondialdehyde levels in the HBD, HMD and HPD groups suggests the occurrence of oxidative stress, which may mediate cardiac dysfunction. However, HCD could not induce oxidative stress in mouse hearts. This suggests that not all forms of long-term dietary long-chain SFA intake share the same mechanisms of myocardial dysfunction.

## 5. Conclusions

Long-term dietary SFA intake can induce cardiac dysfunction without significantly altering metabolic profiles in mice. The altered fatty acid composition of the cardiac phospholipids is dependent on different dietary SFA types. Accumulated total SFA and AA in cardiac phospholipids and increased ceramide levels in the heart may be responsible for SFA intake-induced cardiac dysfunction in mice.

## Figures and Tables

**Table 1:** Fatty acid composition of total lipids in serum of mice fed different high-fat diets ^1^.

	CHD	HBD	HCD	HMD	HPD
**3-month diet**					
14:0	0.09 ± 0.02	0.10 ± 0.01	0.03 ± 0.01 ^#^	0.15 ± 0.01 ^#^	0.05 ± 0.01 ^#^
16:0	4.27 ± 0.50	3.33 ± 0.40 *	3.13 ± 0.23 *	3.40 ± 0.37 *	5.13 ± 0.87 *
16:1 (n-9)	0.81 ± 0.16	0.70 ± 0.06	0.39 ± 0.06 ^#^	0.64 ± 0.17	0.57 ± 0.11 *
18:0	1.17 ± 0.13	1.42 ± 0.29	1.58 ± 0.28	1.02 ± 0.15	1.46 ± 0.30
18:1 (n-9)	2.80 ± 0.40	3.60 ± 0.13 *	2.91 ± 0.24	2.69 ± 0.16	3.91 ± 0.74 ^#^
18:2 (n-6)	2.00 ± 0.38	2.31 ± 0.25	1.64 ± 0.26	2.34 ± 0.32	1.78 ± 0.53
20:4 (n-6)	3.84 ± 0.40	3.23 ± 0.94	3.55 ± 0.68	2.62 ± 0.78	5.19 ± 0.91 *
22:0	0.04 ± 0.01	0.06 ± 0.01	0.07 ± 0.01	0.06 ± 0.01	0.07 ± 0.02 *
22:1	0.06 ± 0.01	0.09 ± 0.05	0.08 ± 0.04	0.07 ± 0.03	0.12 ± 0.06
22:6 (n-3)	0.44 ± 0.06	0.45 ± 0.09	0.45 ± 0.08	0.44 ± 0.15	0.58 ± 0.07
Sum of SFA	5.57 ± 0.57	4.91 ± 0.67	4.80 ± 0.49	4.63 ± 0.53	6.72 ± 1.18 *
Sum of MUFA	3.67 ± 0.53	4.39 ± 0.18 *	3.38 ± 0.28	3.39 ± 0.29	4.60 ± 0.18 *
Sum of PUFA	6.27 ± 0.64	6.04 ± 1.18	5.65 ± 0.97	5.40 ± 1.14	7.55 ± 1.33
**6-month diet**					
14:0	0.07 ± 0.01	0.06 ± 0.01	0.03 ± 0.01 ^#^	0.08 ± 0.02	0.04 ± 0.01 ^#^
16:0	3.16 ± 0.38	3.94 ± 0.29	3.41 ± 0.25	3.55 ± 0.47	4.85 ± 0.70 ^#^
16:1 (n-9)	0.61 ± 0.12	0.56 ± 0.06	0.34 ± 0.04 ^#^	0.47 ± 0.07	0.48 ± 0.08
18:0	0.95 ± 0.14	1.34 ± 0.04 ^#^	1.75 ± 0.20 ^#^	1.03 ± 0.18	1.35 ± 0.12 ^#^
18:1 (n-9)	2.54 ± 0.45	3.75 ± 0.34 *	2.76 ± 0.31	2.58 ± 0.26	3.64 ± 0.69 *
18:2 (n-6)	1.37 ± 0.28	1.78 ± 0.16	1.68 ± 0.29	1.96 ± 0.32	1.59 ± 0.30
20:4 (n-6)	3.51 ± 0.75	4.73 ± 0.20 *	4.52 ± 0.39 *	2.94 ± 0.57	5.34 ± 0.73 ^#^
22:0	0.05 ± 0.01	0.05 ± 0.01	0.07 ± 0.02	0.08 ± 0.02	0.06 ± 0.01
22:1	0.12 ± 0.05	0.12 ± 0.04	0.07 ± 0.03	0.09 ± 0.04	0.08 ± 0.02
22:6 (n-3)	0.25 ± 0.06	0.55 ± 0.03 ^#^	0.42 ± 0.03 ^#^	0.41 ± 0.08 ^#^	0.53 ± 0.11 ^#^
Sum of SFA	4.23 ± 0.51	5.39 ± 0.32	5.25 ± 0.44	4.73 ± 0.66	6.30 ± 0.81 ^#^
Sum of MUFA	3.27 ± 0.54	4.43 ± 0.27 *	3.17 ± 0.36	3.14 ± 0.30	4.19 ± 0.77 *
Sum of PUFA	5.12 ± 1.06	7.06 ± 0.16 *	6.62 ± 0.61 *	5.31 ± 0.94	7.46 ± 0.95 ^#^

^1^ SFA, saturated fatty acid; MUFA, monounsaturated fatty acid; PUFA, polyunsaturated fatty acid; CHD, control high fat diet; HBD, high beef tallow diet; HCD, high cocoa butter diet; HMD, high milk fat diet; HPD, high palm oil diet. Values are mean ± standard deviation (mM), *n* = 5. * *p* < 0.05 and ^#^ *p* < 0.01 versus CHD.

**Table 2:** Fatty acid profile of neutral lipids in hearts of mice fed different high-fat diets ^1^.

	CHD	HBD	HCD	HMD	HPD
**3-month diet**					
14:0	0.22 ± 0.05	0.27 ± 0.02	0.15 ± 0.02	0.38 ± 0.13 ^#^	0.15 ± 0.04
16:0	5.21 ± 0.82	5.11 ± 1.00	3.93 ± 0.63	5.37 ± 1.42	4.36 ± 1.59
16:1 (n-9)	0.94 ± 0.18	0.87 ± 0.17	0.58 ± 0.15	0.89 ± 0.29	0.58 ± 0.13
18:0	2.40 ± 0.31	2.62 ± 0.46	2.34 ± 0.08	2.66 ± 0.45	2.09 ± 0.81
18:1 (n-9)	17.67 ± 2.41	19.46 ± 2.02	17.59 ± 2.84	17.11 ± 4.41	15.81 ± 2.26
18:2 (n-6)	2.40 ± 0.40	2.16 ± 0.27	1.63 ± 0.13	2.76 ± 1.21	1.42 ± 0.29
20:0	0.20 ± 0.01	0.19 ± 0.01	0.20 ± 0.01	0.24 ± 0.02 ^#^	0.20 ± 0.03
20:1	0.52 ± 0.12	0.36 ± 0.03	0.32 ± 0.06	0.50 ± 0.31	0.38 ± 0.06
20:4 (n-6)	0.45 ± 0.06	0.39 ± 0.04	0.42 ± 0.07	0.47 ± 0.11	0.42 ± 0.05
22:0	0.16 ± 0.03	0.17 ± 0.02	0.17 ± 0.00	0.20 ± 0.02 ^#^	0.16 ± 0.01
22:1	5.47 ± 0.50	4.85 ± 0.32	4.59 ± 0.11 *	4.90 ± 0.53	5.60 ± 0.25
22:5	0.39 ± 0.12	0.22 ± 0.08	0.24 ± 0.11	0.25 ± 0.14	0.36 ± 0.11
22:6 (n-3)	0.51 ± 0.09	0.48 ± 0.10	0.52 ± 0.14	0.47 ± 0.32	0.44 ± 0.20
Sum of SFA	8.19 ± 1.14	8.36 ± 1.43	6.80 ± 0.66	9.40 ± 1.22	6.95 ± 2.42
Sum of MUFA	24.60 ± 2.88	25.54 ± 2.11	23.08 ± 3.00	23.41 ± 4.56	22.40 ± 2.59
Sum of PUFA	3.75 ± 0.55	3.24 ± 0.28	2.82 ± 0.40	3.96 ± 1.61	2.63 ± 0.43
**6-month diet**					
14:0	0.19 ± 0.07	0.23 ± 0.08	0.21 ± 0.02	0.27 ± 0.05	0.19 ± 0.05
16:0	5.26 ± 2.11	4.94 ± 0.93	5.40 ± 0.34	5.83 ± 0.60	5.45 ± 1.34
16:1 (n-9)	0.76 ± 0.46	0.77 ± 0.47	0.61 ± 0.09	0.74 ± 0.13	0.53 ± 0.23
18:0	2.48 ± 0.59	2.26 ± 0.94	2.83 ± 0.40	3.34 ± 0.61 *	2.26 ± 0.57
18:1 (n-9)	12.40 ± 3.86	12.08 ± 4.98	10.54 ± 1.90	12.89 ± 2.15	11.63 ± 2.40
18:2 (n-6)	1.82 ± 0.75	1.24 ± 0.62	0.98 ± 0.26	1.29 ± 0.28	1.45 ± 0.78
20:0	0.07 ± 0.03	0.09 ± 0.01	0.08 ± 0.01	0.09 ± 0.01	0.08 ± 0.02
20:1	0.36 ± 0.06	0.31 ± 0.05	0.26 ± 0.04	0.27 ± 0.05	0.34 ± 0.11
20:4 (n-6)	0.39 ± 0.13	0.39 ± 0.14	0.35 ± 0.07	0.34 ± 0.11	0.41 ± 0.06
22:0	0.16 ± 0.02	0.17 ± 0.03	0.18 ± 0.02	0.18 ± 0.03	0.18 ± 0.05
22:1	5.46 ± 0.41	5.04 ± 0.73	5.25 ± 0.67	5.84 ± 0.55	5.63 ± 0.77
22:5	0.36 ± 0.23	0.20 ± 0.10	0.17 ± 0.06 *	0.19 ± 0.08	0.28 ± 0.12
22:6 (n-3)	0.51 ± 0.18	0.32 ± 0.14 *	0.28 ± 0.09 ^#^	0.34 ± 0.13 *	0.49 ± 0.06
Sum of SFA	8.22 ± 2.77	8.08 ± 1.90	8.79 ± 0.71	9.80 ± 1.12	8.23 ± 1.84
Sum of MUFA	18.98 ± 4.39	18.20 ± 5.56	16.66 ± 2.40	19.73 ± 2.32	18.14 ± 2.80
Sum of PUFA	3.09 ± 1.17	2.16 ± 0.86	1.78 ± 0.29 *	2.16 ± 0.31	2.63 ± 0.86

^1^ SFA, saturated fatty acid; MUFA, monounsaturated fatty acid; PUFA, polyunsaturated fatty acid. Values are mean ± standard deviation (μg/mg heart tissue), *n* = 4–8. * *p* < 0.05 and ^#^ *p* < 0.01 versus CHD.

**Table 3:** Fatty acid profile of phospholipids in hearts of mice fed different high-fat diets.

	CHD	HBD	HCD	HMD	HPD
**3-month diet**					
14:0	0.02 ± 0.00	0.04 ± 0.01 ^#^	0.01 ± 0.00	0.05 ± 0.02 ^#^	0.01 ± 0.00
16:0	5.05 ± 0.15	5.35 ± 0.66	4.84 ± 0.17	6.01 ± 0.46 ^#^	5.81 ± 0.16 *
16:1 (n-9)	0.25 ± 0.02	0.28 ± 0.04	0.22 ± 0.04	0.30 ± 0.07	0.16 ± 0.04 *
18:0	5.95 ± 0.30	9.08 ± 0.25 ^#^	10.29 ± 0.77 ^#^	9.82 ± 0.44 ^#^	6.53 ± 0.45
18:1 (n-9)	4.69 ± 0.48	5.60 ± 0.36 ^#^	4.61 ± 0.35	4.57 ± 0.20	4.05 ± 0.35
18:2 (n-6)	1.56 ± 0.35	1.68 ± 0.18	1.60 ± 0.34	2.01 ± 0.36	0.89 ± 0.13 ^#^
20:0	0.18 ± 0.02	0.12 ± 0.02	0.20 ± 0.02	0.18 ± 0.03	0.29 ± 0.25
20:1	0.27 ± 0.03	0.12 ± 0.02 ^#^	0.09 ± 0.01 ^#^	0.10 ± 0.04 ^#^	0.14 ± 0.10 ^#^
20:4 (n-6)	5.19 ± 0.29	6.93 ± 0.87 ^#^	8.17 ± 0.68 ^#^	7.09 ± 0.64 ^#^	5.62 ± 0.68
22:0	0.12 ± 0.01	0.10 ± 0.02	0.14 ± 0.02 *	0.16 ± 0.01 ^#^	0.12 ± 0.01
22:1	0.09 ± 0.03	0.09 ± 0.08	0.08 ± 0.03	0.07 ± 0.02	0.14 ± 0.06
22:5	0.53 ± 0.06	0.65 ± 0.13	0.60 ± 0.08	1.24 ± 0.10 ^#^	0.79 ± 0.21 *
22:6 (n-3)	7.81 ± 0.20	8.79 ± 0.83	8.03 ± 0.70	9.93 ± 0.65 ^#^	7.68 ± 0.71
Sum of SFA	11.32 ± 0.28	14.69 ± 0.89 ^#^	15.49 ± 0.74 ^#^	16.22 ± 0.59 ^#^	12.75 ± 0.73 ^#^
Sum of MUFA	5.31 ± 0.50	6.09 ± 0.40 ^#^	4.99 ± 0.35	5.03 ± 0.26	4.49 ± 0.24 *
Sum of PUFA	15.09 ± 0.46	18.05 ± 1.62 ^#^	18.40 ± 1.34 ^#^	20.27 ± 0.85 ^#^	14.99 ± 0.95
**6-month diet**					
14:0	0.02 ± 0.00	0.02 ± 0.01	0.02 ± 0.00	0.02 ± 0.01	0.01 ± 0.01
16:0	4.89 ± 0.76	4.55 ± 0.18	4.67 ± 0.46	4.85 ± 0.33	5.72 ± 0.64 ^#^
16:1 (n-9)	0.28 ± 0.14	0.19 ± 0.02	0.19 ± 0.04	0.18 ± 0.02	0.17 ± 0.06 *
18:0	5.37 ± 0.67	7.76 ± 0.36 ^#^	8.43 ± 0.74 ^#^	7.26 ± 0.60 ^#^	6.68 ± 0.51 ^#^
18:1 (n-9)	4.00 ± 0.65	4.32 ± 0.35	3.55 ± 0.37	3.22 ± 0.25 *	4.04 ± 0.50
18:2 (n-6)	1.01 ± 0.17	0.81 ± 0.19	0.68 ± 0.13 *	0.84 ± 0.27	0.87 ± 0.24
20:0	0.15 ± 0.03	0.12 ± 0.02 *	0.18 ± 0.02	0.12 ± 0.02 *	0.17 ± 0.01
20:1	0.16 ± 0.05	0.09 ± 0.03 ^#^	0.07 ± 0.01 ^#^	0.06 ± 0.01 ^#^	0.11 ± 0.05 ^#^
20:4 (n-6)	4.48 ± 0.69	6.76 ± 0.79 ^#^	6.42 ± 0.93 ^#^	5.56 ± 0.87 *	6.11 ± 0.72 ^#^
22:0	0.10 ± 0.02	0.10 ± 0.01	0.12 ± 0.02 *	0.10 ± 0.01	0.11 ± 0.02
22:1	0.10 ± 0.03	0.10 ± 0.03	0.13 ± 0.05	0.08 ± 0.01	0.12 ± 0.04
22:5	0.61 ± 0.14	0.80 ± 0.09 *	0.64 ± 0.12	0.86 ± 0.17 ^#^	0.67 ± 0.06
22:6 (n-3)	7.96 ± 1.95	7.85 ± 0.85	8.74 ± 1.11	8.12 ± 0.70	8.34 ± 1.79
Sum of SFA	10.52 ± 1.37	12.55 ± 0.37 ^#^	13.41 ± 1.19 ^#^	12.36 ± 0.80 ^#^	12.70 ± 1.00 *
Sum of MUFA	4.53 ± 0.81	4.70 ± 0.36	3.95 ± 0.38	3.54 ± 0.26 *	4.43 ± 0.55
Sum of PUFA	14.07 ± 2.49	16.21 ± 0.51	16.48 ± 1.93	15.38 ± 1.12	16.00 ± 1.44

Values are mean ± standard deviation (μg/mg heart tissue), *n* = 4–8. * *p* < 0.05 and ^#^ *p* < 0.01 versus CHD.

## Supplementary Materials

The following are available online at www.mdpi.com/2072-6643/10/1/106/s1, Table S1: Fatty acids composition of experimental diets, Table S2: Body weight and heart weight of mice at 3 and 6 months of feeding regimen, Table S3: Metabolic parameters of mice fed high fat diets for 3 months, Table S4: Relevant parameters for cardiac function results from mice fed high fat diets.
