# Supplementary material for: Dietary Fatty Acids Alter Lipid Profiles and Induce Myocardial Dysfunction without Causing Metabolic Disorders in Mice
Source: Nutrients. 2018 Jan 19;10(1):106. doi: 10.3390/nu10010106 (PMC5793334; doi:10.3390/nu10010106)
Supplement: Supplementary file 1 [file nutrients-10-00106-s001.zip › Supplementary File 2.pdf]

Supplementary Tables

Table S1 Fatty acids composition of experimental diets*

	CHD	HBD	HCD	HMD	HPD
**Total Fatty Acids, g/kg diet**					
Total	200	200	200	200	200
Saturated fat	178	99	114	121	102
Monounsaturated fat	3	80	67	60	78
Polyunsaturated fat	19	21	19	20	19
**Fatty Acid Profile, % of total fatty acids**					
Saturated fat	89.1	49.5	57.0	60.4	51.3
Monounsaturated fat	1.6	40.2	33.7	29.8	39.0
Polyunsaturated fat	9.4	10.3	9.3	9.8	9.7
4:0 (Butyric acid)	0.0	0.0	0.0	3.0	0.0
6:0 (Hexanoic acid)	1.8	0.0	0.0	2.1	0.0
8:0 (Caprylic acid)	60.8	0.0	0.0	1.0	0.0
10:0 (Capric acid)	24.9	0.0	0.0	1.8	0.0
12:0 (Lauric acid)	0.0	0.0	0.0	2.8	0.0
14:0 (Myristic acid)	0.0	3.0	0.1	10.7	1.1
16:0 (Palmitic acid)	0.8	24.2	24.2	24.5	45.3
16:1 (Palmitoleic acid)	0.0	3.1	0.3	1.7	0.0
18:0 (Stearic acid)	0.3	19.8	31.8	11.7	4.7
18:1 (Oleic acid)	1.6	36.3	33.4	26.9	39.0
18:2 (Linoleic acid)	9.4	9.4	9.3	9.3	9.4
18:3 (Linolenic acid)	0.0	0.5	0.0	0.5	0.3

CHD, control high fat diet; HBD, high beef tallow diet; HCD, high cocoa butter diet; HMD, high milk fat diet; HPD, high palm oil diet.

* Data were supplied by Harlan Laboratories.

Table S2 Body weight and heart weight of mice at 3 and 6 months of feeding regimen.

	CHD	HBD	HCD	HMD	HPD
**3-month diet**					
BW (g)	32.39 ± 2.10	31.12 ± 3.02	29.99 ± 1.81	28.35 ± 5.77	38.16 ± 4.46*
HW (mg)	119.9 ± 7.9	122.6 ± 14.0	116.6 ± 7.9	115.6 ± 17.1	138.7 ± 9.5
HW/BW	3.72 ± 0.45	3.97 ± 0.59	3.89 ± 0.24	4.18 ± 0.78	3.41 ± 0.12
**6-month diet**					
BW (g)	35.27 ± 2.53	35.81 ± 1.42	35.52 ± 4.49	42.77 ± 3.75*	39.66 ± 2.02
HW (mg)	139.6 ± 14.7	128.4 ± 9.1	126.5 ± 20.6	152.3 ± 7.7	137.0 ± 9.7
HW/BW	3.95 ± 0.18	3.59 ± 0.23	3.56 ± 0.35	3.59 ± 0.49	3.46 ± 0.29

BW, body weight; HW, heart weight; CHD, control high fat diet; HBD, high beef tallow diet; HCD, high cocoa butter diet; HMD, high milk fat diet; HPD, high palm oil diet. Values are mean ± standard deviation, n= 5. *P<0.05 *versus* CHD.

Table S3 Metabolic parameters of mice fed high fat diets for 3 months

	**CHD**	**HBD**	**HCD**	**HMD**	**HPD**
SBP (mmHg)	96.50 ± 3.66	101.77 ± 7.26	100.69 ± 2.89	103.69 ± 4.86	107.43 ± 18.44
DBP (mmHg)	70.04 ± 5.02	71.45 ± 4.51	72.00 ± 3.05	75.64 ± 2.87	79.23 ± 16.32
Hear rate (bpm)	710.8 ± 31.8	726.8 ± 27.4	722.6 ± 37.5	688.8 ± 60.9	722.3 ± 49.8
FBGL (mmol/L)	7.0 ± 1.4	7.6 ± 2.7	7.1 ± 1.5	7.8 ± 2.1	5.5 ± 1.1

SBP, systolic blood pressure; DBP, diastolic blood pressure; FBGL, fasting blood glucose level; CHD, control high fat diet; HBD, high beef tallow diet; HCD, high cocoa butter diet; HMD, high milk fat diet; HPD, high palm oil diet. Values are mean ± standard deviation, n = 5.

Table S4 Relevant parameters for cardiac function results from mice fed high fat diets.

	**CHD**	**HBD**	**HCD**	**HMD**	**HPD**
**3-month diet**					
LVPWT (%)	41.6 ± 3.3	35.1 ± 2.8*	34.2 ± 4.3*	30.7 ± 3.8*	35.6 ± 2.5*
LVIDd (mm)	3.61 ± 0.24	3.61± 0.27	3.55 ± 0.39	3.67 ± 0.43	3.71 ± 0.51
LVIDS (mm)	1.81 ± 0.24	2.36 ± 0.24*	2.25 ± 0.33*	2.39 ± 0.45*	2.48 ± 0.37*
IVRT (msec)	15.1 ± 1.1	17.1 ± 0.9*	17.5 ± 1.0*	18.2 ± 0.9*	17.2 ± 1.3*
Heart rate (bpm)	422 ± 46	430 ± 29	438 ± 30	466 ± 12	441 ± 28
**6-month diet**					
LVPWT (%)	40.2 ± 2.6	31.0 ± 2.1*	32.8 ± 2.8*	27.6 ± 1.1*	33.2 ± 1.9*
LVIDd (mm)	3.82 ± 0.22	3.84 ± 0.41	3.94 ± 0.46	4.09 ± 0.51	4.02 ± 0.49
LVIDs (mm)	1.97 ± 0.10	2.69 ± 0.28*	2.64 ± 0.47*	2.76 ± 0.33*	2.80 ± 0.50*
IVRT (msec)	15.8 ± 1.8	19.2 ± 1.8*	18.6 ± 1.5	19.8 ± 2.0*	19.4 ± 1.4*
Heart rate (bpm)	468 ± 19	469 ± 25	445 ± 53	457 ± 37	457 ± 48

LV, Left Ventricular; LVIDd, LV internal dimensions at diastole; LVIDs, LV internal dimensions at systole; LVPWT, LV posterior wall thickening; IVRT, isovolumetric relaxation time. n=5, $^{*}P<0.05$ *versus* CHD.
