# Supplementary material for: Dietary Fatty Acids Alter Lipid Profiles and Induce Myocardial Dysfunction without Causing Metabolic Disorders in Mice
Source: Nutrients. 2018 Jan 19;10(1):106. doi: 10.3390/nu10010106 (PMC5793334; doi:10.3390/nu10010106)
Supplement: Supplementary file 1 [file nutrients-10-00106-s001.zip › Supplementary File 1.pdf]

Online Supporting Material

Methods

Blood pressure measurement

Blood pressure was measured on mice after they had been fed on high-fat diets for 3 months, using a tail-cuff method. Briefly, mice were gently restrained in a holder and warmed to 37°C by a heating platform under the holder. Animals were acclimated in the environment for 5-10 minutes after occlusion cuffs and pulse transducers (CODA^{TM} system, Kent Scientific Corporation, USA) were placed on the tail. The values of at least 10 readings for each mouse were used for blood pressure and heart rate analysis.

Intraperitoneal glucose tolerance and insulin resistance tests

For intraperitoneal glucose tolerance test (IPGTT), 16-h fasted mice were given a glucose load (2 g/kg,i.p.). Blood samples were taken from the tail vein at 0, 15, 30, 45, 60, 120 minutes after glucose injection, and blood glucose was measured using a glucose meter (LifeScan, CA, USA). For intraperitoneal insulin tolerance test (IPITT), insulin was injected intraperitoneally (0.75 U/kg) into 4-h fasted mice, and blood glucose was measured at time points of 0, 15, 30, 45 and 60 minutes post injection.

Cardiomyocyte cross-sectional area assessment

The cardiomyocyte cross-sectional area were assessed as described in our previous study [1]. Briefly, hearts were excised, washed with saline solution,and placed in 10% formalin. Hearts were then cut transversely close to theapex to visualize the left ventricle and right ventricle. For cardiomyocyte cross-sectional area, sections were stained for membranes with fluorescein isothiocyanate–conjugated wheat germ agglutinin (WGA; Invitrogen) and for nuclei with DAPI. A single cardiomyocyte was measured with an image quantitative digital analysis system (NIH Image version 1.6). The outline of at least 200 cardiomyocytes was traced in each heart.

Online Supporting Material

Echocardiography

Cardiac function was evaluated by echocardiography at the end of each diet period as described previously [2]. Briefly, animals were lightly anesthetized with inhaled isoflurane (1%) and imaged in a warm handling platform using a 40-MHz linear array transducer (MS-550D, FUJIFILM, VisualSonics, Toronto, Canada) attached to a preclinical ultrasound system (Vevo 2100, FUJIFILM, VisualSonics) with nominal in-plane spatial resolution of 40 μm (axial) × 80 μm (lateral). M-mode and 2-D parasternal short-axis scans (133 frames/second) at the level of the papillary muscles were used to assess changes in left ventricle (LV) end-systolic inner diameter (LVIDs), LV end-diastolic inner diameter (LVIDd), LV posterior wall thickness in end-diastole (LVPWd) and end-systole (LVPWs), and fractional shortening (FS%), as we recently described [3]. The LV posterior wall thickening (LVPWT) is calculated by using the following formula: LVPWT (%) = 100 × [(LVPWs – LVPWd)/LVPWd] [4]. To assess diastolic function, we obtained apical four chamber view of the left ventricle. The pulsed wave Doppler measurements were obtained in the apical view with a cursor at mitral valve inflow: maximal early (E) and late (A) transmitral velocities in diastole. The ratio of E over A was used to evaluate diastolic function.

Serum fatty acids analysis with gas chromatography

The total serum fatty acids were extracted as described previously [5]. In brief, mice serum samples (50 μl) were mixed with 1.5 ml chloroform-methanol (2:1, v/v), containing 0.01% butylated hydroxytoluene as antioxidant, and with 20 μg methyl nonadecanoate (Sigma, USA) as an internal standard. The mixture was votexed for 1 minute and centrifuged at 12000 g for 10 minutes. The chloroform phase was transferred to a clean glass vial using a glass Pasteur pipette and evaporated to dryness under a stream of nitrogen. The residues were transmethylated to fatty acid methyl esters (FAMEs) by incubation with 1 ml 3 M methanolic HCl for 2 hours at 80°C. The

reaction was stopped by adding saturated sodium chloride solution (1 ml) and the mixture was allowed to cool to room temperature. The lipid methyl esters were extracted with hexane (3 x using 2 ml), pooled and dried under nitrogen. Finally, samples were reconstituted in 500μl hexane and analyzed using Agilent 7890 gas chromatography (GC) equipped with a CTC-PAL autosampler. Samples (1 μl) were injected (splitless mode; injector temperature = 270°C) onto a CP-Si18 column (ChromPak; 15 m x 0.25 mm ID; 0.25 μm film thickness) and eluted with the following temperature gradient: initial column temperature at 100°C, held for 2 min, followed by a 10°C/min ramp to a final temperature of 300°C. The final temperature was held for 3 min for a total run time of 25 min. Compounds were detected with a flame ionization detector (FID) at 300°C. Nitrogen was used as a carrier gas at 2 ml/min. Individual FAMEs were identified by comparison to an authentic standard mixture of FAMEs (Supelco), and comparison of mass spectrometry (MS) generated on a Varian 3800 GC coupled with a 220 ion trap MS operated under the same conditions (except He was used as a carrier gas at 1 ml/min). GC data were analyzed using Agilent ChemStation software (Version B.04.02 SP1).

Fatty acids analysis for heart tissues

Frozen heart tissues (10~15 mg) were homogenized in pre-cooled 0.9% sodium chloride solution, and total lipids were extracted according to the method of Folch*et al* [5]. Three internal standards (methyl nonadecanoate, 1, 2 - dipentadecanoyl - sn - glycerol - 3 - phosphoethanolamine, and N - heptadecanoyl - D - erythro - sphingosine) were added at 20 μg each before the extraction. Lipid fractionation was performed according to previous reports with some modification [6, 7]. Briefly, the total lipid extracts were resuspended in 200 μl chloroform and loaded onto a Supelclean LC-NH_2 column (Supelco, LC-NH_2 1-ml tubes, Sigma, USA), which had been previously conditioned with 2 ml hexane. The samples were allowed to adsorb to the column matrix by gravity.

Online Supporting Material

Different lipid subclasses were eluted as follows: 2 ml chloroform-isopropanol (2:1, v/v) to elute neutral lipids (NL); 2 ml diisopropyl ether-acetic aicd (98:2, v/v) to elute free fatty acids and 3 ml methanol to elute phospholipids (PL). The fraction containing free fatty acids was discarded. The fatty acid composition of PL and NL fractions was analyzed by GC using the protocol described above for serum fatty acids.

Ceramide detection by mass spectrometry

Ceramides were eluted in the NL fraction during the tissue lipid fractionation process, according to the report from Bodennec*et al* [7]. The NL fraction was evaporated to dryness under nitrogen and resuspended in 200 µl methanol containing 1 mM ammonium formate and 0.2% formic acid and analyzed using an Agilent 1260 HPLC coupled with an Agilent 6230A TOF MS, using a modified protocol based on that previously described in the literature [8]. Samples (10µl) were injected onto a Zorbax-Extend C-18 column (Rapid Resolution HT, 2.1 x 50 mm, 1.8 µm; Agilent Technologies) and eluted with a linear gradient of 1 mM NH_4 acetate in MeOH (containing 0.2% formic acid; Solvent B) in 2 mM NH_4 acetate (aqueous, containing 0.2% formic acid; Solvent A) according to the following program: 2-minute isocratic elution at 80% Solvent B in Solvent A, followed by a 10-minute gradient to 99% Solvent B in Solvent A, and 15 minutes isocratic at 99% Solvent B in Solvent A, at a flow rate of 0.25 ml/min. After return to start conditions (80% Solvent B in Solvent A), the column was equilibrated to 7 minutes before the next injection. The column was housed in a thermostatic compartment set at 30°C. The eluent was monitored at 203 nm prior to introduction to the TOF MS detector through a Dual ESI source. Hot (325°C) N_2 drying gas (12 l/min; 45 psi) was used to nebulize the LC eluent. A VCap of 4500V and fragmentor voltage of 125V was used. Reference ions (121.050873 and 922.009798) were introduced into the TOF at a constant rate through the second nebulizer of the Dual ESI, at 5 psi. Data were collected in the

100-1500 m/z range and analyzed using Agilent Mass Hunter software (Version B05.00). 2-Amino-1,3-dihydroxyoctadecene ceramides were identified and quantified on the basis of their exact masses (C-16, 537.5121; C-18, 565.5434; C-20, 593.5747; C-24:1, 647.6216; C-24, 649.6373) in positive ion mode. Quantitation was based on normalization to the internal standard (C-17 ceramide) and a calibration curve prepared from authentic C-17 ceramide.

References of Methods

1. Li, J.; Zhu, H.; Shen, E.; Wan, L.; Arnold, J. M.; Peng, T., Deficiency of rac1 blocks NADPH oxidase activation, inhibits endoplasmic reticulum stress, and reduces myocardial remodeling in a mouse model of type 1 diabetes. *Diabetes* **2010,** 59, (8), 2033-42.

2. Respress, J. L.; Wehrens, X. H., Transthoracic echocardiography in mice. *J Vis Exp* **2010**, (39).

3. Ma, J.; Wei, M.; Wang, Q.; Li, J.; Wang, H.; Liu, W.; Lacefield, J. C.; Greer, P. A.; Karmazyn, M.; Fan, G. C.; Peng, T., Deficiency of Capn4 gene inhibits nuclear factor-kappaB (NF-kappaB) protein signaling/inflammation and reduces remodeling after myocardial infarction. *J Biol Chem* **2012,** 287, (33), 27480-9.

4. Gao, S.; Ho, D.; Vatner, D. E.; Vatner, S. F., Echocardiography in Mice. *Curr Protoc Mouse Biol* **2011,** 1, 71-83.

5. Folch, J.; Lees, M.; Sloane Stanley, G. H., A simple method for the isolation and purification of total lipides from animal tissues. *J Biol Chem* **1957,** 226, (1), 497-509.

6. Gerson, A. R.; Brown, J. C.; Thomas, R.; Bernards, M. A.; Staples, J. F., Effects of dietary polyunsaturated fatty acids on mitochondrial metabolism in mammalian hibernation. *J Exp Biol* **2008,** 211, (Pt 16), 2689-99.

7. Bodennec, J.; Koul, O.; Aguado, I.; Brichon, G.; Zwingelstein, G.; Portoukalian, J., A procedure for fractionation of sphingolipid classes by solid-phase extraction on aminopropyl cartridges. *J Lipid Res* **2000,** 41, (9), 1524-31.

8. Bielawski, J.; Szulc, Z. M.; Hannun, Y. A.; Bielawska, A., Simultaneous quantitative analysis of bioactive sphingolipids by high-performance liquid chromatography-tandem mass spectrometry. *Methods* **2006,** 39, (2), 82-91.
